# Unraveling the biochemical and kinetic mechanisms of potassium solubilization from biotite by a *Burkholderia* strain

**DOI:** 10.1371/journal.pone.0360380

**Published:** 2026-10-09

**Authors:** Zihan Dong, Lei Zhang, Yue Miao, Yun Chen, Lihong Liu, Lufan Qiu

**Affiliations:** 1 School of Earth Science, Northeast Petroleum University, Daqing, Heilongjiang, China; 2 The sixth oil production plant, Daqing OILFIELD Limited Company, Daqing, Heilongjiang, China; IIT Roorkee: Indian Institute of Technology Roorkee, INDIA

## Abstract

Potassium solubilizing bacteria (KSB) represent a sustainable strategy for enhancing soil K bioavailability through the dissolution of K-bearing minerals, thereby facilitating nutrient cycling. However, the multifaceted biochemical mechanisms driving mineral weathering remain partially understood. The present study explores the K solubilization characteristic and mechanisms of KSB by analyzing the organic acids they metabolize and their interactions with mineral surfaces. Over 30 d, the strain S1 classified to genus *Burkholderia* achieved a maximum soluble K^+^ concentration of 39.8 mg/L, representing a 4.5-fold increase over abiotic controls. Mechanistic analysis revealed a synergistic “dual-attack” strategy: continuous acidification (pH 6.42 to 3.82) promoted proton-driven K^+^ release, while the dominant secretion of citric and malic acids facilitated ligand-mediated complexation of structural cations, destabilizing the aluminosilicate framework. Additionally, the temporal accumulation of polar and acidic amino acids likely served as biogenic regulators, enhancing bacteria-mineral adhesion and interface interactions. Scanning electron microscopy corroborated these chemical findings, revealing extensive surface etch pits and edge exfoliation. These results suggest that the synergistic interaction of proton attack, ligand-mediated chelation, and amino acid-assisted micro-environment regulation drives mineral weathering. This study provides a comprehensive structural framework for KSB-mediated nutrient mobilization, highlighting the potential of *Burkholderia* as an efficient bio-fertilizer for potassium-deficient soils.

## Introduction

Potassium (K) is a vital macronutrient essential for plant physiological processes, including enzyme activation, osmotic regulation, and photosynthesis [1]. Despite its abundance in the Earth’s crust as a component of silicate minerals, over 90% of mineral K remains in a chemically recalcitrant form inaccessible to plants [2]. Intensive agricultural practices and excessive tillage have led to severe soil K depletion, necessitating a reliance on synthetic chemical fertilizers [3,4]. However, the long-term application of these fertilizers often results in soil structural damage, reduced organic matter, and significant environmental footprints [5–7], which pose substantial risks to agricultural sustainability [8,9].

KSB represent a promising bio-based strategy to mitigate this crisis [10–12]. By accelerating the natural weathering of K-bearing minerals, KSB not only optimize nutrient cycling but also improve plant resilience to abiotic stress [13–16]. Various genera, including *Bacillus*, *Rhizobium*, and *Burkholderia*, have been documented for their ability to dissolve silicate rocks and enhance K availability in diverse rhizosphere environments [17–21]. Emerging screening techniques, such as the use of pH-indicator dyes (e.g., bromothymol blue), have significantly improved our capacity to isolate efficient KSB from complex soil samples [22,23].

Despite the well-established benefits of KSB in promoting plant growth, the underlying biochemical mechanisms responsible for mineral K solubilization remain poorly characterized [24,25]. It is widely hypothesized that KSB mobilize K primarily through acidolysis and ligand-mediated chelation [26,27]; however, these processes are rarely quantified in a coupled experimental system. Furthermore, while the role of organic acids in weathering is recognized, the contribution of other potential biogenic agents, in particular, extracellular amino acids and their role in modulating the bacteria-mineral interface, remains an overlooked dimension of KSB-mediated mineral degradation.

In this study, we employed a *Burkholderia* strain isolated from Northeast China to elucidate the precise mechanisms of K solubilization from biotite. By integrating long-term kinetic monitoring, quantitative analysis of extracellular metabolites (organic acids and amino acids), and high-resolution Scanning electron micrographs (SEM), this study provides three key insights: (1) the thermodynamic driving forces of proton and ligand-promoted dissolution; (2) the auxiliary regulatory role of extracellular amino acids in bacterial-mineral interaction; and (3) a comprehensive kinetic and structural framework for biotite weathering. These results not only deepen our understanding of microbe-mineral interactions at the biochemical level but also provide a robust theoretical foundation for the development of highly efficient bio-fertilizers.

## Materials and methods

### Soil sampling

In this study, soil sample W1 was collected from rice fields in Mudanjiang City, China, at a depth of 5–25 cm. Three replicate samples were collected, homogenized, and immediately sealed in sterilized sampling bags to prevent exogenous microbial contamination. The samples were stored in an ice chest and analyzed within 24 h of collection. The physicochemical properties of the soil sample were presented in Supporting information S1 File.

### Screening, isolation and identification of KSB

#### Culture substrates.

Selective medium: agar 20.0 g, CaCO_3_ 0.1 g, FeCl_3_ 0.005 g, MgSO_4_ •7H_2_O 0.5 g, Na_2_HPO_4_•12H_2_O 2.0 g, sucrose 5.0 g, ultrapure water 1000 mL, biotite 1.0 g, pH 7.0, The medium was autoclaved at 121°C for 20 min.

Modified medium was prepared by adding 0.25% (w/v) bromothymol blue to the selective Medium., The medium was autoclaved at 121°C for 20 min.

LB medium: agar 20.0 g, NaCl 10.0 g, tryptone 10.0 g, ultrapure water 1000mL, yeast extract 5.0 g, pH 7.0, The medium was autoclaved at 121°C for 20 min.

The compositions of the selective medium, modified medium, and LB medium were modified from the original formulations to enhance the activity of microorganisms and promote vigorous bacterial growth [28].

Biotite was bought from Xingtang County Xinlei Mineral Powder Processing Factory, China. Other chemicals above were bought from Sinopharm Chemical Reagent Co., Ltd, China.

#### Isolation and screening of KSB from soil samples in the laboratory.

A 10 g soil sample was added into 100 mL sterile saline solution (0.8%NaCl) and mixed thoroughly by shaking conical flask on table concentrator for 30 mins at room temperature to obtain a bacterial suspension. The suspension was then inoculated into 100 mL selective medium, previously sterilized at 121 °C for 20 min, for bacterial incubation and enrichment by shaking conical flask at 28 ± 2 °C for 3 d. This process was repeated three times to obtain a solution containing the target bacteria.

After being diluted 5 times, 0.2 mL of each degree suspension was spread over sterilized modified medium in triplicate. The inoculated plates were incubated in a constant-temperature incubator at 28 ± 2 °C for 3 d. Large, fast-growing and slimy colonies were selected and inoculated into modified medium for purifying. The plates were then incubated at 28 ± 2 °C for 7 d while the colonies showing a transparent zone around them were considered as KSB. Screened bacterial strains were subjected to Gram staining according to standard protocols for preliminary characterization [29]. The purified colonies placed in modified medium were stored at 4 °C for later use.

#### Identification of bacterial strains.

The screened bacterial strains were subjected to a battery of biochemical assays to assess their metabolic capabilities. These tests encompassed catalase test, citrate utilization test, indole production test, gelatin liquefaction test, methyl red test, nitrate reduction test, oxidase test, sugar fermentation test, starch hydrolysis test, and Voges-Proskauer test. Test results were compared against standard biochemical profiles of known KSB (e.g., *Bacillus* spp., *Pseudomonas* spp.) as referenced in Bergey’s Manual of Systematic Bacteriology. Isolates were tentatively identified to the genus level based on consistent physiological and biochemical characteristics.

A molecular identification approach based on 16S rRNA gene sequencing was employed to determine the taxonomic affiliation of the screened bacterial strains [30]. Genomic DNA was extracted from the purified colonies using Genomic DNA Purification Kit of Sang Biotech, following the manufacturer’s protocol. The universal primers 27F (AGAGTTTGATCATGGCTCAG) and 1492R (TAGGGTTACCTTGTTA

CGACTT) were used for the amplification of 16S rRNA gene via polymerase chain reaction (PCR). The PCR reaction mixture (50 μL total volume) consists of 5.0 μL 10 × Ex Taq buffer, 4.0 μL 2.5 mM dNTP Mix, 2.0 μL 10 μM Primer 27F, 2.0 μL 10 μM Primer 1492R, 0.5 μL 5 U Ex Taq polymerase, 2.0 μL template DNA, and 34.5 μL ultrapure water. The PCR cycling conditions were as follows: initial denaturation at 94°C for 3 min; 30 cycles of denaturation at 94°C for 30 s, annealing at 54°C for 30 s, and extension at 72°C for 90 s; followed by a final extension at 72°C for 10 min.

PCR products were analyzed by 1% agarose gel electrophoresis at 150 V and 100 mA for 20 min to verify the amplicon size (~1500 bp). Gels were visualized using a gel imaging system. Amplicons were purified and sequenced by Sang Biotech. The obtained 1241-bp 16S rRNA gene sequence was analyzed using the 16S-based Identification tool at EZBioCloud. The phylogenetic tree was constructed in MEGA software (version 7.0) using the neighbor-joining method based on the closest type strains identified by EZBioCloud [31].

### Mineral preparation

Biotite, a K-bearing silicate mineral, was specifically selected as the model mineral to validate the activity of KSB in this study for two key reasons supported by both mineralogical and microbiological evidence. At first, biotite contains ~7–9% K_2_O (w/w) with K exclusively located in the interlayer sites of 2:1 phyllosilicate, where K^+^ is coordinated by 12 oxygen atoms in a hexagonal ring, resulting in lower hydration energy (−322 kJ/mol) compared to K-feldspar (−380 kJ/mol). This makes interlayer K⁺ more bioavailable to microbial attack. In addition, Multiple KSB strains have been repeatedly shown to preferentially extract K from trioctahedral micas (biotite) rather than feldspar or illite, with dissolution rates 3–8 times higher in biotite systems. Therefore, biotite serves as both a sensitive indicator and a theoretically sound substrate for assessing KSB K-solubilizing efficacy.

Biotite was ground and passed through a 1-mm sieve. The elemental composition of the biotite was confirmed by energy-dispersive X-ray spectroscopy (Figure S4.A in S1 File). The specific surface area was determined to be 1.15 m^2^/g, and the particle size distribution after sieving through a 1-mm mesh is provided in Table S4.B in S1 File. The sieved powder was washed with 0.01 mol/L HCl to eliminate exchangeable bases cations, and washed with deionized water until the wastewater became neutral. Finally, the biotite was dried in an oven at 120 ± 5°C to reach a constant weight.

### Experimental design and incubation

The leaching experiments were conducted in 250 mL Erlenmeyer flasks containing 100 mL of modified medium (previously sterilized at 121°C for 20 min and supplemented with 1 g of biotite). All treatments (Treatment, CK1, CK2, and CK3) were performed in independent biological triplicates (n = 3 × 5 separate Erlenmeyer flasks per treatment), with destructive sampling at each time point to avoid disturbing the system. For each biological replicate, leachate samples were analyzed in technical triplicates to account for instrumental and pipetting variability.

**Treatment (live bacteria + biotite):** Flasks were inoculated with 1% (v/v) of bacterial suspension (≈10^8^ CFU/mL, OD_600_ ≈ 0.8).
**CK1 (abiotic control):** Biotite+ sterile medium (no inoculum).
**CK2 (metabolites control):** Biotite + cell-free fermentation supernatant. The supernatant was prepared by culturing the strain in the same medium (without biotite) for 7 d, followed by centrifugation (10000 × g, 10 min, 4°C) and filtration through a 0.22 μm sterile membrane. The sterility of CK2 and all abiotic controls was confirmed by plating aliquots on LB agar plates and incubating at 30 °C for 7 d, with no microbial growth observed.
**CK3 (background control):** Bacterial culture liquid without biotite (to subtract any K released from the inoculum itself).

All groups were incubated in a constant-temperature shaker at 30°C, pH 6.5, and 130 rpm shaking speed for 30 d.

### Sampling and analytical methods

Samples were destructively harvested at 5, 10, 15, 20, 25, and 30 d for liquid parameters (three independent flasks per time point per treatment). For SEM, Biotite samples were collected every 15 d (0, 15, 30) from separate parallel flasks to avoid disturbing the system.

#### Determination of available K content.

Culture supernatants were centrifuged (10,000 × g, 10 min) and filtered (0.22 μm). The soluble K content was determined by atomic absorption spectrophotometry (AAS). The instrument used was a PERSEE TAS-990F atomic absorption spectrophotometer equipped with a K hollow cathode lamp, with the wavelength set at 766.5 nm and a slit width of 0.7 nm.

#### pH measurement.

The pH of the culture supernatant was measured immediately using a digital pH meter (REX PHSJ-5T) calibrated with standard buffers.

#### Preparation of organic acid standard solutions.

Individual stock solutions (1 g/L) of oxalic acid, tartaric acid, malic acid, acetic acid, and citric acid were prepared by accurately weighing 0.05 g of each standard, dissolving in ultrapure water, and diluting to 50 mL in volumetric flasks. A mixed standard solution (0.20 g/L for each acid) was prepared by combining 40 mL of each individual stock solution and diluting to 200 mL with ultrapure water. Working standard solutions at concentrations of 40, 80, 120, 160, and 200 mg/L were prepared by diluting 10, 20, 30, 40, and 50 mL of the mixed standard solution to 50 mL with ultrapure water, respectively. All solutions were filtered through 0.22 μm organic membrane filters and stored at 4°C until analysis.

#### High-performance liquid chromatography (HPLC) conditions.

HPLC analysis was performed on a Shimadzu LC-20AD system equipped with an Ultimate AQ-C18 column (4.6 mm × 250 mm, 5 μm). The column temperature was maintained at 30°C, the injection volume was 10 μL, and detection was carried out at 210 nm with a UV detector. This wavelength was selected because it is optimal for the sensitive detection of low-molecular-weight organic acids and amino acids due to the strong absorbance of their carboxyl groups in the low UV range. The mobile phase consists of (A) 20 mM disodium hydrogen phosphate buffer (pH 2.60, adjusted with phosphoric acid) mixed with methanol (99:1, v/v) and (B) methanol. Both mobile phases were filtered through 0.45 μm membranes and ultrasonically degassed for 15 min before use. Retention times were determined using the individual 1 g/L standard solutions, and peak identities were confirmed with the mixed standard solution. Calibration curves were constructed using peak areas of the working standard solutions (40–200 mg/L).

#### Organic acids.

An 8 mL aliquot of supernatant was transferred to a 10 mL centrifuge tube and centrifuged at 11,000 rpm for 8 min. Subsequently, 0.75 mL of the supernatant was mixed with 0.75 mL of acetonitrile in a centrifuge tube and centrifuged again at 11,000 rpm for 8 min. The resulting supernatant was filtered through a 0.22 μm organic membrane filter. Finally, 0.5 mL of the filtrate was transferred into a vial for HPLC analysis using a disposable syringe.

#### Amino acids.

A 10 mL aliquot of supernatant was transferred to a centrifuge tube and centrifuged at 10,000 rpm for 10 min. Subsequently, 5 mL of the supernatant was mixed with an equal volume (5 mL) of 100% (w/v) trichloroacetic acid (TCA) to achieve a final concentration of 50% (w/v) TCA. The mixture was ultrasonicated at 50 ± 2 °C for 2 h. The mixture was allowed to stand for 24 h at room temperature, followed by centrifugation at 11,000 rpm for 8 min. Four mL of the resulting supernatant was collected, evaporated to dryness under a stream of nitrogen (or in a rotary evaporator at appropriate temperature), and redissolved in 5 mL of 0.02 mol/L hydrochloric acid (HCl). The solution was filtered through a 0.22 μm organic membrane filter. Finally, 0.5 mL of the filtrate was transferred into a vial using a disposable syringe for HPLC analysis.

#### Scanning electron microscopy.

At each time point, biotite particles were carefully collected and gently washed with phosphate-buffered saline (PBS, pH 7.2) to remove loosely attached cells, fixed in 2.5% (v/v) glutaraldehyde (4°C, 12 h). Subsequently, samples were dehydrated through a graded ethanol series (30%, 50%, 70%, 90%, and 100%) to preserve structural integrity. A metal film with a thickness of 15 nm was sputter-coated onto the sample surface using a gold-palladium alloy to enhance conductivity, and the samples were imaged using a scanning electron microscope (JSM-6510, operating at 15 kV). Images were captured at multiple magnifications to visualize surface etching, bacterial attachment, and biofilm formation.

### Statistical analysis

The experimental data were analyzed using Python (version 3.9) with the scipy.stats library. Pearson correlation analysis was performed to assess the relationship between soluble K⁺ concentration and pH. Differences among treatments at each time point were evaluated by one-way ANOVA followed by Tukey’s post-hoc test (p < 0.05). All experiments were performed in three independent biological replicates (n = 3 separate Erlenmeyer flasks per treatment per time point). Error bars in all figures represent the standard deviation of the mean from these biological replicates.

## Results

### Physiological and biochemical characteristics of KSB

The physiological and biochemical characteristics of the KSB strain are as follows. Experimental outcomes revealed positive reactions for catalase test, indole production test, gelatin liquefaction test, and sugar fermentation test, and negative reactions for citrate utilization test, methyl red test, nitrate reduction test, oxidase test, starch hydrolysis test, and Voges-Proskauer test. The overall biochemical profile is consistent with members of the genus *Burkholderia* within a polyphasic taxonomic framework.

In this study, the screened colonies were subjected to Gram staining and observed under a microscope, with results presented in Fig 1. The cells of the strain were clearly dispersed, appearing as short rods and stained red, confirming that it is a Gram-negative bacterium.

**Fig 1:**
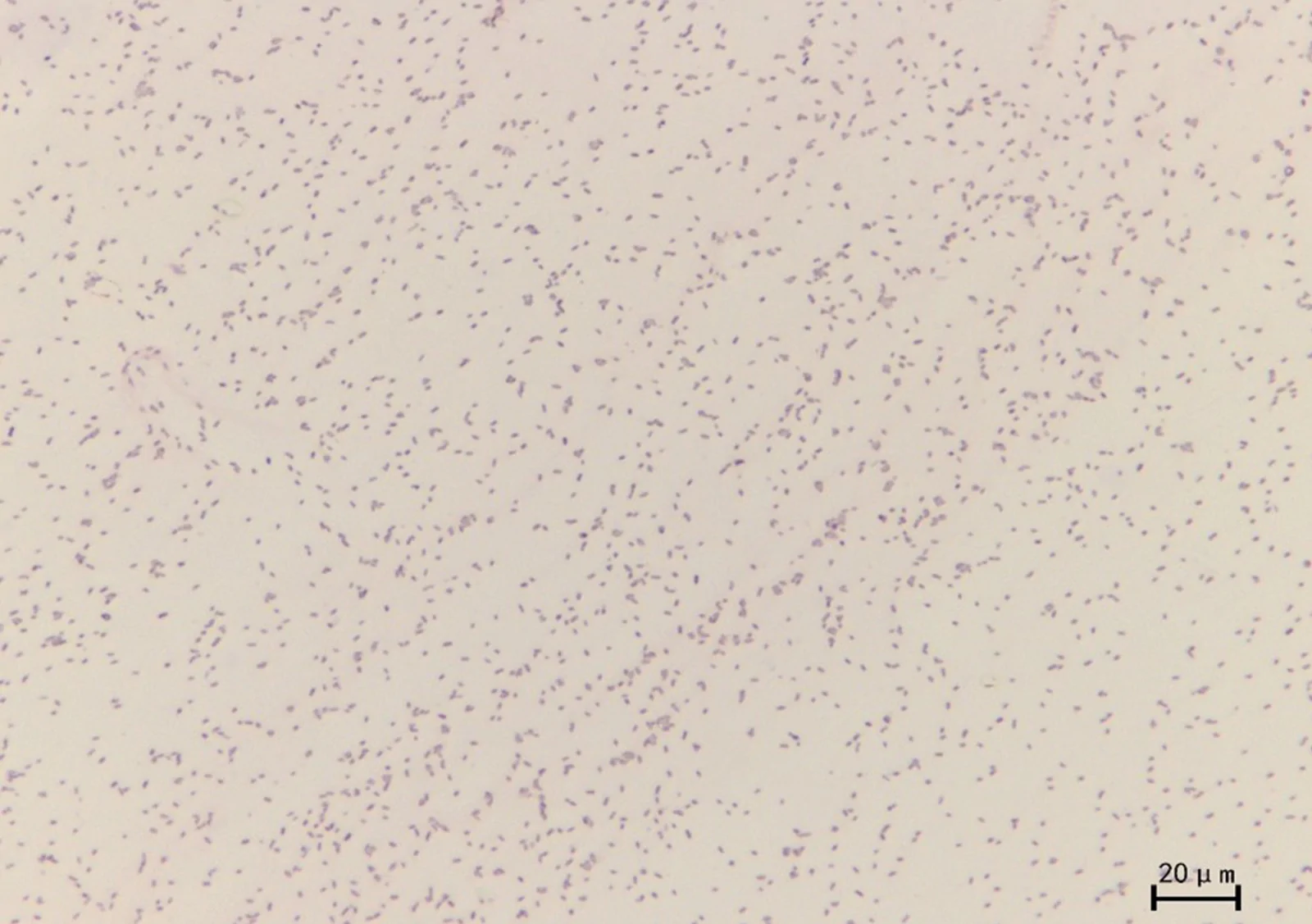
Gram staining of KSB strain S1 cells.

The morphology of KSB strain S1 was examined by SEM, as shown in Fig 2. The cells appeared as short rods (0.8–1.2 μm in length), with wrinkled surfaces, flagella, and no spores.

**Fig 2:**
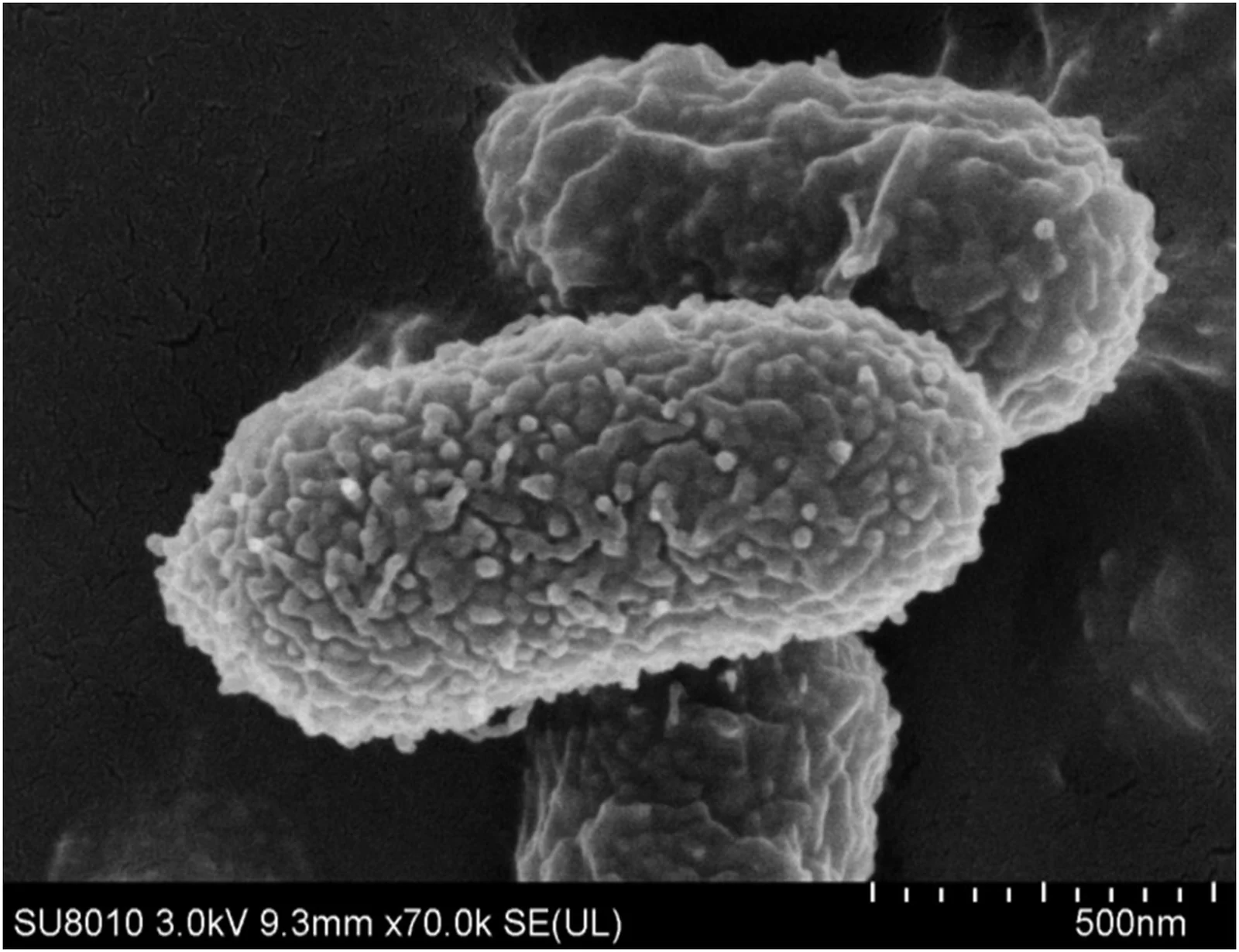
Scanning electron micrograph of KSB strain S1.

Based on the 16S rRNA gene sequence of strain S1, taxonomic identification was performed using the 16S-based ID tool at EZBioCloud. The analysis identified *Burkholderia cepacia* ATCC 25416 as the closest type strain, with a sequence similarity of 99.76% (The figure is presented in the Supporting information S3 in S1 File). The phylogenetic tree was constructed using MEGA 7.0 software with the neighbor-joining method based on the closest reference type strains, as shown in Fig 3. Strain S1 clustered with members of the genus *Burkholderia* on the same evolutionary branch. The sequence similarity of 99.76% clearly exceeds the 95% threshold generally accepted for genus-level assignment. Therefore, strain S1 belongs to the genus *Burkholderia.*

**Fig 3:**
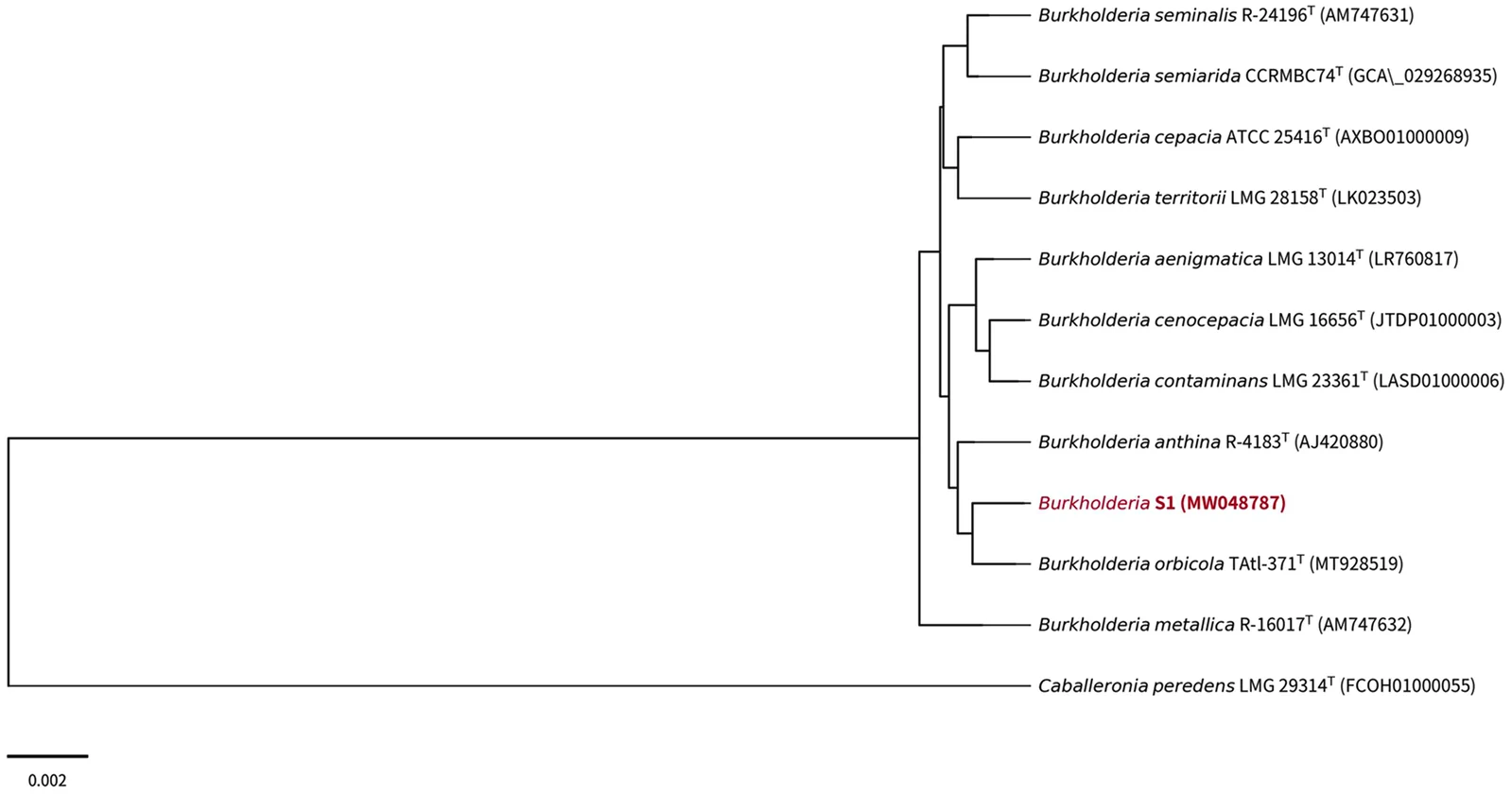
Phylogenetic tree of 16S rRNA gene sequences of KSB strain S1 and type strains of closely related Burkholderia species.

### Dynamic effects of KSB on K release from biotite

The continuous bio-weathering capacity of the strain S1 on K-bearing minerals was evaluated over a 30 d incubation period (Fig 4). The results demonstrated a pronounced enhancement of K⁺ release in the presence of the live KSB strain (Treatment) compared to all respective controls.

**Fig 4:**
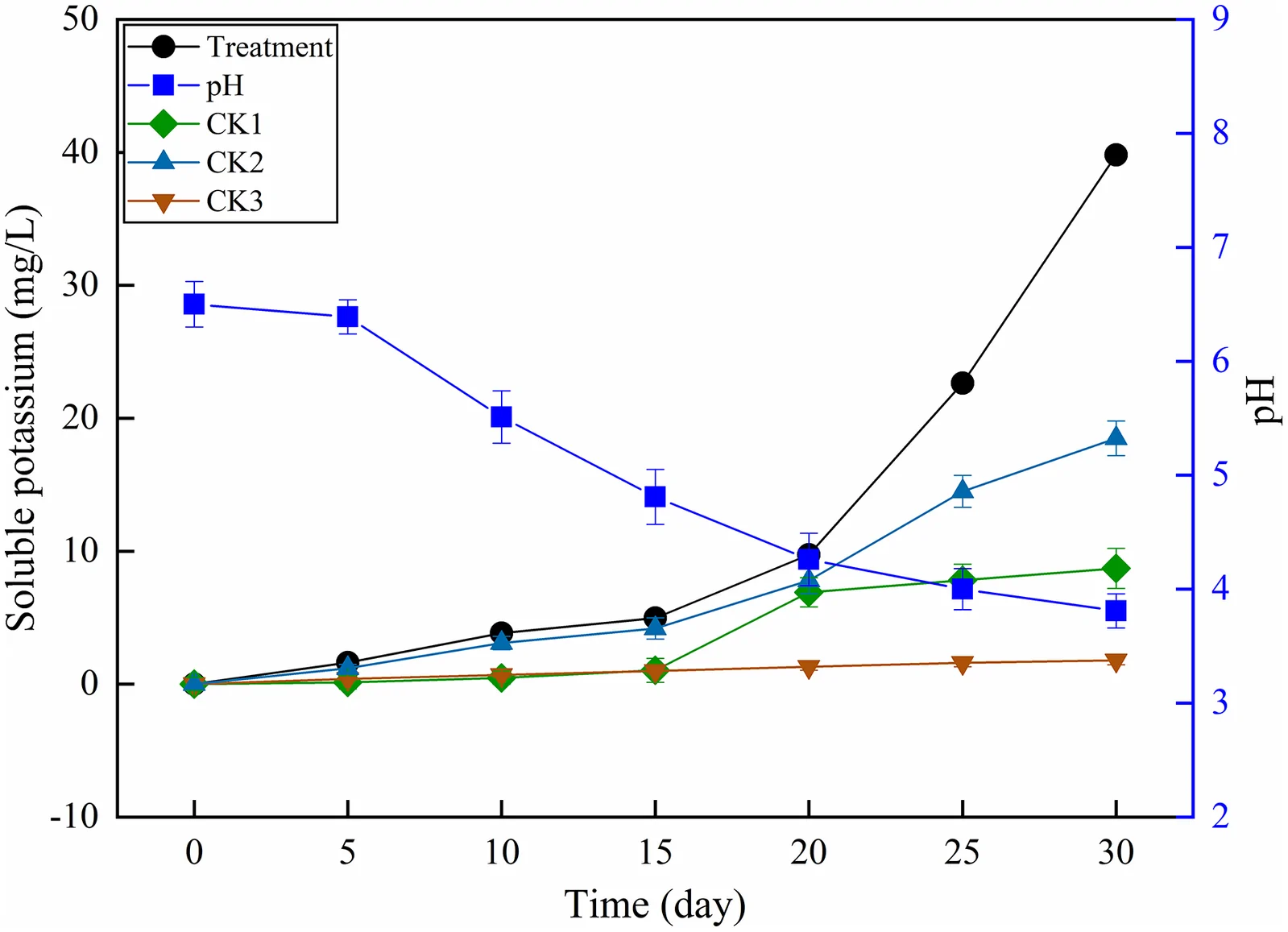
Dynamics of soluble K concentration and pH during the 30-d biotite weathering experiment. “Treatment” represents biotite inoculated with live KSB; “CK1” serves as the abiotic control (biotite + sterile medium); “CK2” evaluates indirect leaching (biotite + cell-free supernatant); and “CK3” monitors background K⁺ (culture medium only). Error bars indicate standard deviations of biological replicates.

One-way ANOVA revealed that the soluble K concentration differed significantly among the four treatments (F = 7.32, p = 0.0002). Tukey’s HSD post-hoc test further showed that the treatment group released significantly more K⁺ than CK1 (p = 0.0065) and CK3 (p = 0.0002), while the difference with CK2 was not statistically significant (p = 0.2178). Specifically, the soluble K concentration in the treatment group exhibited a distinct biphasic release pattern: a gradual accumulation phase from 0 to 15 d (reaching ~ 4.97 mg/L), followed by a phase of rapid, near-exponential release. The maximum soluble K⁺ concentration peaked at approximately 39.8 mg/L on 30 d.

In contrast, the abiotic background release from biotite in the sterile medium (CK1) remained minimal, plateauing at roughly 8.71 mg/L by the end of the experiment. Furthermore, the bacterial culture medium itself (CK3) maintained negligible K⁺ levels (1.8 mg/L) throughout, eliminating the possibility of background nutrient interference.

The inclusion of the cell-free fermentation supernatant (CK2) effectively isolates the role of continuous bacterial metabolism. The CK2 group produced moderate mineral dissolution, and the soluble K⁺ concentration reached 18.5 mg/L at 30 d. This value was significantly lower than that obtained with live cells of KSB strain S1. By 30 d, the K⁺ solubilization efficiency of the treatment group was approximately 4.5 times higher than that of the abiotic control (CK1) and 2.1 times higher than the metabolite-only control (CK2). Concurrently, the system pH experienced a steady decline from an initial value of 6.4 to 3.8, which temporally coincided with the accelerated K⁺ release phase, strongly indicating a proton-promoted dissolution mechanism.

### Evolution of the physicochemical environment and secretion of typical organic acids

To elucidate the chemical mechanisms driving the accelerated K⁺ release, the dynamic changes in pH and the secretion profiles of low-molecular-weight organic acids (LMWOAs) were systematically monitored.

As illustrated in Fig 5, the continuous microbial metabolism in the treatment group induced a substantial and steady decline in system pH, dropping from an initial 6.42 to 3.82 over the 30d incubation. This pronounced acidification phase directly coincided with the exponential surge in soluble K ⁺ . Pearson correlation analysis (Table 1) confirmed a significant negative correlation between system pH and soluble K⁺ concentration in the treatment group (*r* = −0.799, *p* < 0.05). This statistical evidence strongly supports a proton-promoted dissolution mechanism, where the continuous generation of H⁺ by live KSB actively attacks the mineral lattice. In contrast, the pH of the cell-free supernatant control (CK2) remained relatively stable, fluctuating only marginally (5.5–5.8), resulting in a positive correlation with K⁺ release (*r* = 0.902, *p* < 0.01). The significant final pH difference (ΔpH ≈ 2.0) between the treatment group and the CK2 group underscores that continuous biogenic proton generation is a primary driving force, significantly outperforming the static acidity of the initial metabolites.

**Fig 5:**
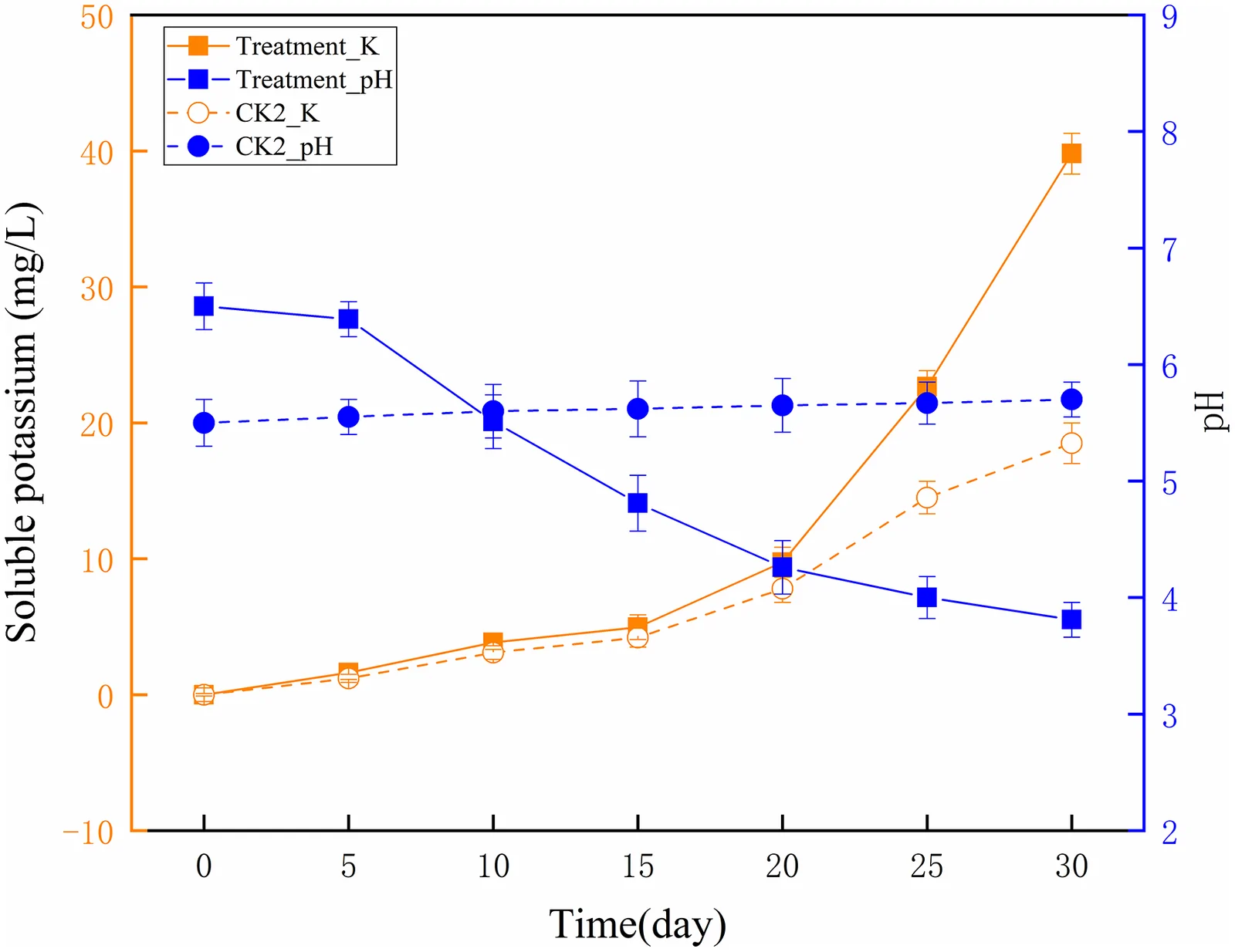
Dynamic changes in system pH and corresponding soluble K⁺ concentrations.

**Table 1:** Pearson correlation analysis between soluble K⁺ concentration and system pH in the Treatment and CK2 groups over the 30-d incubation.

Group	*r* (Pearson)	*p*-value	Interpretation
Treatment	−0.799	<0.05	Significant negative correlation
CK2	0.902	<0.01	Significant positive correlation

Note: All analyses were performed on biological triplicates; error propagation is reported as standard deviation in figures.

Beyond proton attack, the continuous exudation of specific LMWOAs further facilitates mineral decomposition through ligand complexation. Analysis of the bacterial exudates during the early fermentation stage (1–5 d, Fig 6) revealed the consistent secretion of four primary organic acids: citric acid, malic acid, tartaric acid, and ethanedioic (oxalic) acid. The organic acid pool was overwhelmingly dominated by citric acid (accounting for 50–53% of the total detected acids) and malic acid (41–45%).

**Fig 6:**
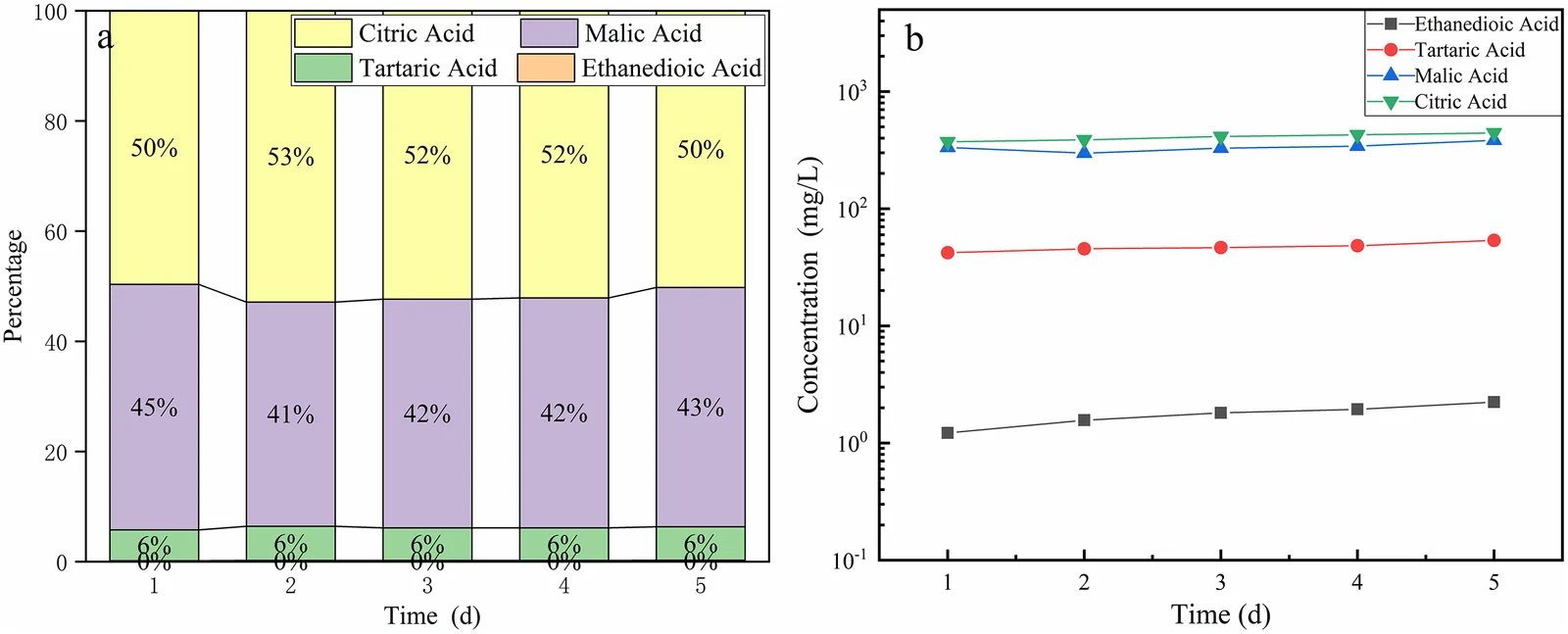
Secretion profiles of LMWOAs by the KSB strain. (A) Relative abundance (percentage) showing the dominance of organic acids. (B) Absolute concentrations (log scale) of the detected organic acids over time.

Quantitative analysis demonstrated that the absolute concentrations of these dominant acids were maintained at exceptionally high levels, with citric and malic acids fluctuating between approximately 300–450 mg/L. Tartaric acid was detected at moderate concentrations (~40–50 mg/L), while ethanedioic acid remained a minor component (1–2 mg/L). Both citric and malic acids are powerful multidentate ligands containing multiple carboxyl and hydroxyl functional groups. Their high abundance in the system provides a strong thermodynamic capacity to chelate structural cations (e.g., Al^3+^, Fe^2+^/Fe^3+^, Mg^2+^) on the biotite surface. This ligand-promoted dissolution synergistically couples with the aggressive proton attack, effectively destabilizing the aluminosilicate framework and accelerating the concomitant release of interlayer K^+^.

### Dynamic secretion characteristics of amino acids

In addition to low-molecular-weight organic acids, the exudation profiles of amino acids were closely monitored during the initial 5 d mineral weathering phase. Amino acids not only reflect the physiological metabolic state of the bacteria but can also serve as auxiliary biogenic weathering agents [32].

As visually depicted in the time-course heatmap (Fig 7) and supported by quantitative data (Table 2), the total concentration of extracellular amino acids exhibited a nearly 10-fold exponential increase, surging from 2.92 nmol/mL on 1 d to 27.52 nmol/mL by 5 d. This pronounced upsurge indicates robust bacterial proliferation and active extracellular metabolism observed during the mineral-weathering process.

**Fig 7:**
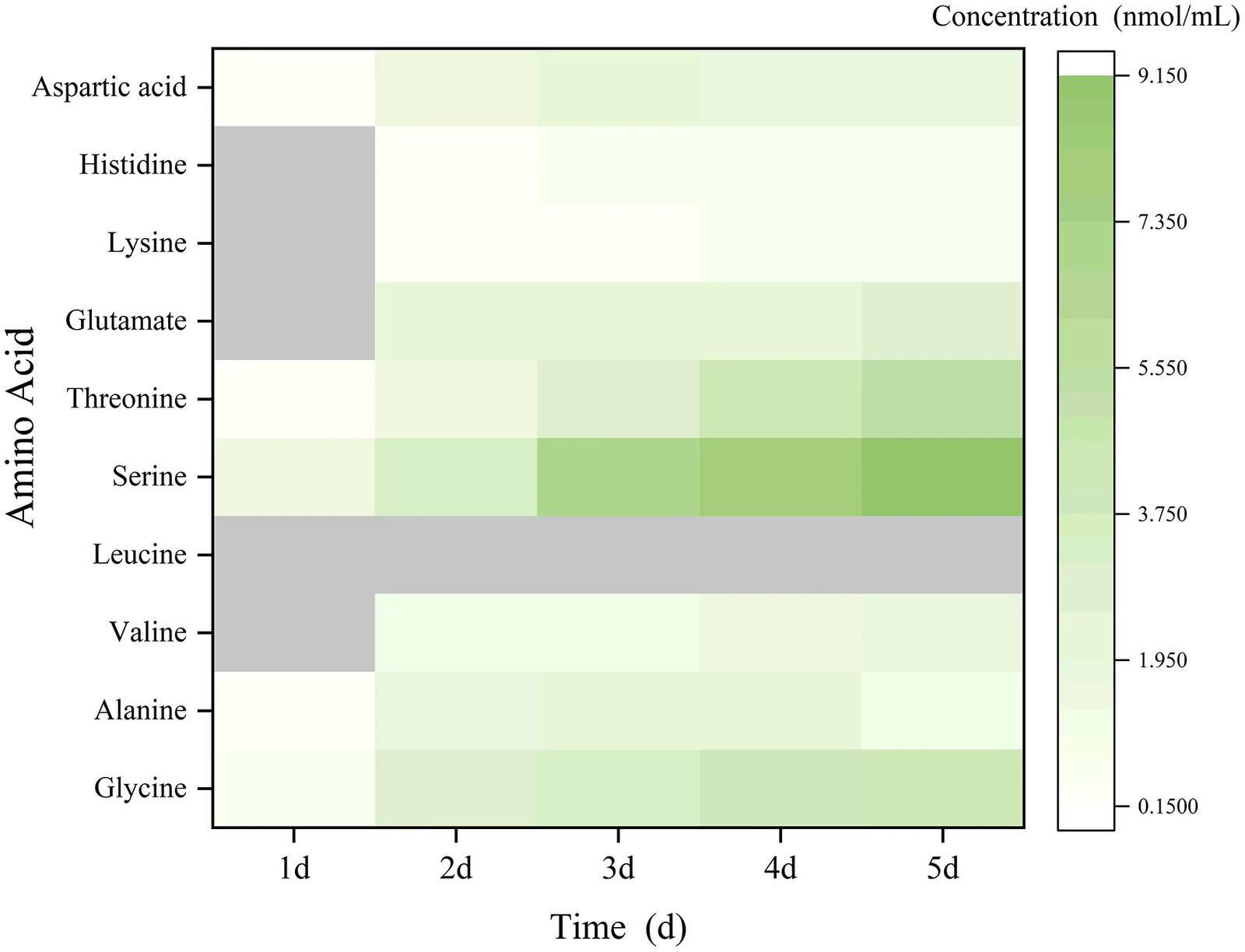
Heatmap illustrating the dynamic temporal succession and accumulation of extracellular amino acids secreted by the KSB strain during the initial 5-d of biotite weathering. The color gradient from white to dark green represents increasing concentrations (nmol/mL), while orange indicates undetected levels (below the limit of detection).

**Table 2:** Quantitative analysis of extracellular amino acid concentrations (nmol/mL) over a 5-d incubation period.

Amino Acid	1d	2d	3d	4d	5d
Glycine	0.68	2.65	3.42	3.86	4.33
Alanine	0.39	1.66	2.13	2.25	1.19
Valine	-^a^	1.11	1.32	1.59	1.76
Leucine	–	–	–	–	–
Serine	1.36	3.39	6.85	7.91	9.14
Threonine	0.33	1.47	2.85	4.13	5.49
Glutamate	–	2.11	2.32	2.43	2.66
Lysine	–	0.41	0.45	0.49	0.54
Histidine	–	0.35	0.46	0.58	0.65
Aspartic acid	0.16	1.65	2.16	1.88	1.76
Total	2.92	14.8	21.96	25.12	27.52

^a^“-” = not detected / below the limit of detection.

A temporal succession in the amino acid composition was clearly observed. During the first 24 h, the exudate was characterized by low diversity, with only five detectable amino acids. However, by 2 d, specific amino acids such as valine, glutamate, lysine, and histidine emerged and began to accumulate. Throughout the 5-d monitoring period, serine (increasing from 1.36 to 9.14 nmol/mL), threonine (0.33 to 5.49 nmol/mL), and glycine (0.68 to 4.33 nmol/mL) constituted the dominant secreted species. Notably, serine and threonine are characterized by highly polar hydroxyl (-OH) side chains, which may enhance the affinity and adhesion of bacterial exudates to the polarized mineral surface.

Furthermore, the significant accumulation of acidic amino acids warrants particular attention. Glutamate (glutamic acid) was initially undetectable but emerged strongly on 2 d (2.11 nmol/mL) and accumulated continuously, while aspartic acid concentrations increased more than 10-fold by 3 d (peaking at 2.16 nmol/mL) before stabilizing. Both glutamate and aspartic acid possess additional carboxyl (-COOH) functional groups on their side chains [33,34]. The substantial secretion of these specific acidic and polar amino acids suggests their potential capability to act as auxiliary multidentate ligands. By synergizing with the dominant organic acids (citric and malic acids), these amino acids can provide further complexation sites for structural cations (e.g., Al^3+^, Fe^3+^), thereby accelerating the destabilization of the biotite lattice and the concomitant release of K⁺ [35]. This is consistent with the role of plant growth-promoting lead-tolerant bacteria in bio-remediation and enhancing plant performance under heavy metal stress [36].

### Evolution of biotite surface weathering micro-morphology

To provide direct physical evidence of mineral lattice destruction and to corroborate the chemical and biological weathering mechanisms discussed above, the surface micro-morphology of biotite was tracked using SEM over the 30-d incubation period.

As shown in the time-course SEM image matrix (Fig 8), the pristine biotite particles on 0 d exhibited a typical phyllosilicate morphology. The unweathered particles were characterized by smooth and flat surfaces, sharp angular edges, and highly intact, tightly packed basal cleavage planes.

**Fig 8:**
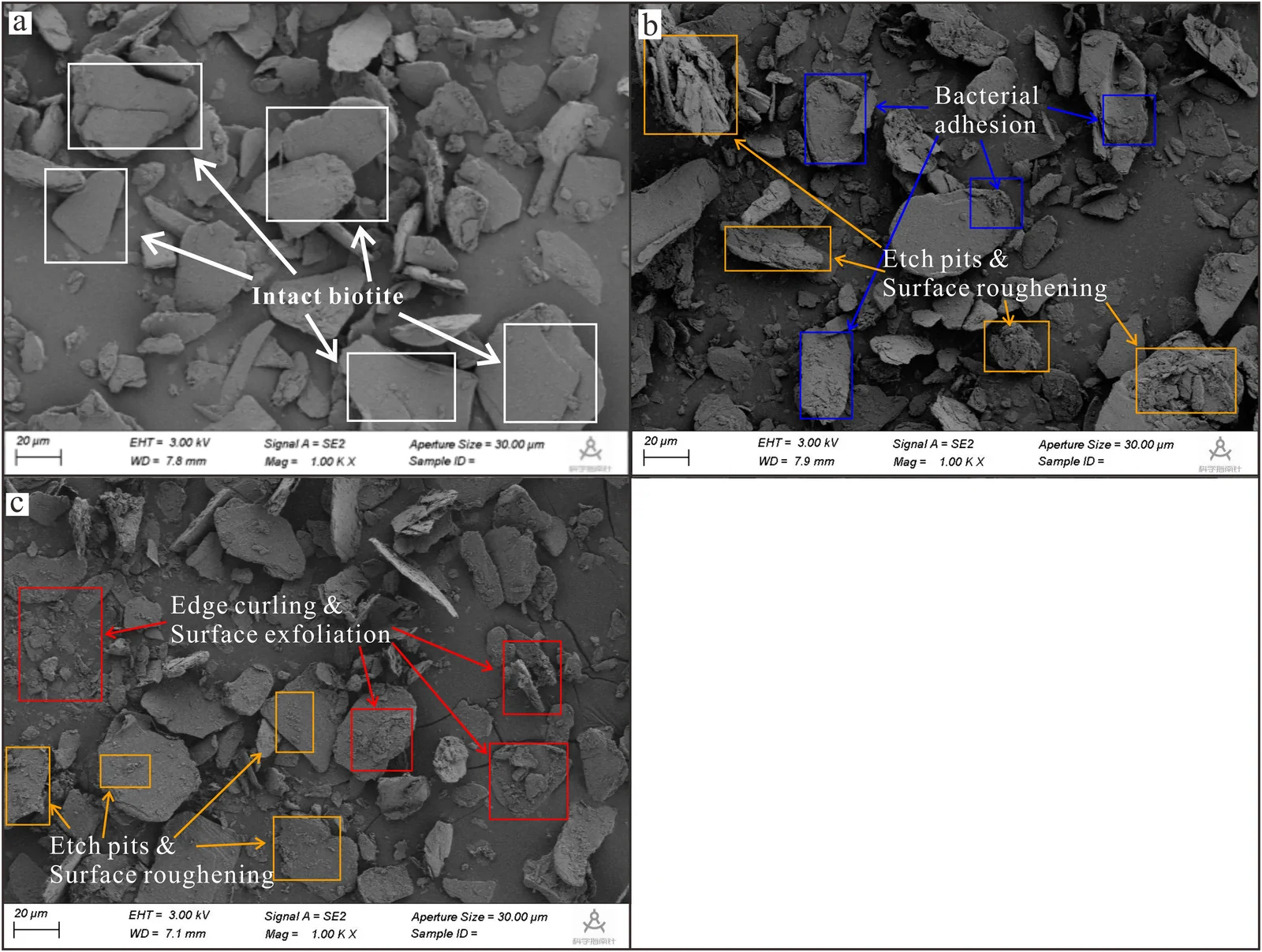
Scanning electron micrographs showing the micromorphological evolution and bacteria-biotite interactions during the 30 d bioweathering process. (a) **0 d**(Control): Intact biotite morphology showing clean basal planes and sharp, well-defined mineral edges (white rectangle). (b) **15 d:** Initial weathering phase showing intimate bacterial adhesion (blue rectangle). The etch pits and surface roughening on the background substrate (orange rectangle) indicates the formation of a biogenic layer/biofilm interface. Higher-resolution SEM images are shown in S5A and S5B Figs in S1 File. (c) **30 d:** Peak degradation stage characterized by severe structural disintegration. Significant edge curling and surface exfoliation (red rectangle) demonstrate the cumulative effect of the “dual-attack” mechanism. Original SEM micrographs of the samples from this study. Imaging services were provided by SciCompass. Reproduced with permission from SciCompass under a CC BY license, original copyright 2025.

By 15 d, corresponding to the onset of the rapid K⁺ release phase and the significant drop in system pH, early-stage weathering features became distinctly visible. The formerly smooth mineral surfaces exhibited noticeable roughening and the initial formation of irregular etch pits. Furthermore, the edges of the biotite flakes began to show signs of mechanical weakening, characterized by slight fraying and the separation of the uppermost structural layers. These morphological alterations provide direct visual evidence of aggressive proton attack on the aluminosilicate framework.

By 30 d, the biotite structural integrity was severely compromised, aligning with the peak accumulation of multidentate LMWOAs (citric and malic acids) and auxiliary acidic amino acids. High-magnification observations revealed pervasive dissolution features. The etch pits had expanded and coalesced, while the distinct layered structure underwent extensive edge exfoliation and curling. The stripping of these silicate sheets significantly increased the specific surface area, thereby exposing more interlayer K⁺ to the solvent. Concurrently, distinct biogenic features were observed, including the adhesion of bacterial cells and the precipitation of amorphous secondary mineral phases on the weathered surfaces. This intimate bacteria-mineral contact, facilitated by the secretion of polar amino acids (e.g., serine, threonine) identified in **Results** section, creates localized microenvironments with concentrated weathering agents, effectively driving the continuous and irreversible breakdown of the biotite lattice.

## Discussion

### Synergistic mechanism of proton and ligand-promoted dissolution

The pronounced increase in soluble K⁺ accompanied by a significant pH decline (Fig 1) suggests a complex interplay between chemical and biological factors [37–39]. Our results demonstrate that the *Burkholderia* strain effectively mobilizes K from biotite through a synergistic “dual-attack” strategy involving protons (H⁺) and LMWOAs.

The initial phase of dissolution is primarily driven by proton-promoted weathering [40]. The high concentration of H⁺ (pH < 4.0) facilitates the exchange of interlayer K⁺ with protons (Equation 2), a process confirmed by the early appearance of surface roughening in SEM images (Fig 8a, b) [41,42]. However, proton attack alone often leads to the formation of a Si-Al-rich depleted layer that can armor the mineral surface and slow down further dissolution. This is where the ligand-promoted mechanism becomes critical. The dominance of citric and malic acids (constituting over 90% of total LMWOAs, Fig 3) provides multidentate ligands capable of forming stable surface complexes with framework cations such as Al^3+^ and Fe^3+^ [43]. By stripping these structural ions from the biotite lattice, LMWOAs effectively compromise the crystalline integrity, preventing the formation of a protective passivating layer and exposing new reactive sites for continuous K⁺ release.

### Kinetic insights: Shifting the thermodynamic equilibrium

The efficacy of the *Burkholderia* strain can be further elucidated through the general dissolution rate equation (1) [44–46]:


$$\begin{array}{c} \begin{array}{c} R=k[x]\left(\text{1}-\frac{Q}{K}\right) \end{array} \end{array}$$ (1)

where $R=\;\frac{d_{Q}}{d_{t}}$ is the K⁺ release rate, [x] is the remaining mineral concentration, Q is the cumulative amount of K⁺ released at time *t*, K is the equilibrium maximum release, and k is the apparent dissolution rate constant. This model allows us to distinguish between direct and indirec*t* contributions of bacterial metabolites.

To provide the requested quantitative analysis, non-linear least-squares regression was applied to the 30-d cumulative K⁺ release data using the integrated pseudo-first-order form of Equation (2):


$$\begin{array}{c} Q(t)=Q_{max}\left(\text{1}-e^{-kt}\right) \end{array}$$

Physically reasonable upper bounds were imposed on $Q_{max}$ (60 mg/L for the treatment and 20 mg/L for the control) based on the total potassium content of biotite and the maximum observed release. The fitted parameters are:

Treatment: $Q_{max\;}=\text{6}\text{0}\pm \text{2}\text{1}\text{5}$ mg/L, k=0.01773±0.07861 d^−1^, $R^{\text{2}}=\text{0}.\text{6}\text{6}\text{3}\text{7}$;
CK1: $Q_{max\;}=\text{2}\text{0}\pm \text{6}\text{2}.\text{7}\text{8}$ mg/L, k=0.01581±0.06004 d^−1^, $R^{\text{2}}=\text{0}.\text{7}\text{5}\text{8}\text{3}$.

The apparent dissolution rate constant k of the treatment group was higher than that of the control, demonstrating that inoculation with the *Burkholderia* strain significantly enhanced K^+^ release from biotite. This kinetic enhancement is consistent with the observed 4.57-fold higher cumulative release at 30 d in the treatment group compared with the control.

Notably, the treatment group exhibited clear acceleration of K^+^ release between day 20 and day 30, a pattern better captured by a supplementary power-law model ($Q={\text{0}.\text{0}\text{0}\text{0}\text{9}\text{4}t}^{\text{3}.\text{1}\text{3}\text{3}},\;R^{\text{2}}=\text{0}.\text{9}\text{9}\text{1}\text{3}$) that reflects microbially driven sigmoidal kinetics.

In the early stages, the system is far from equilibrium (Q/K < 1), and the dissolution rate R is directly proportional to the activity of protons and ligands [x]. However, as cations accumulate in the supernatant, the system approaches a saturated state (Q/K ≈ 1), which would theoretically suppress the dissolution rate. Our data suggest that the KSB strain overcomes this thermodynamic bottleneck through complexation-induced undersaturation. The organic acid anions (e.g., citrate and malate) act as chemical “sinks,” sequestering free Fe^2+^, Mg^2+^, and Al^3+^ into soluble organo-metallic complexes (Equation 2) [47].


$$\begin{array}{c} {Fe}^{\text{2}+}+{C_{\text{6}}H_{\text{5}}O_{\text{7}}}^{\text{3}-}\to {{Fe(C}_{\text{6}}H_{\text{5}}O_{\text{7}})}^{-}+{\text{3}H}^{+} \end{array}$$ (2)

This sequestration significantly lowers the ion activity product (Q), effectively pulling the reaction away from equilibrium and maintaining a high thermodynamic driving force (1-Q/K) for irreversible biotite breakdown.

The treatment-group k value falls within the range reported for KSB treated biotite systems (typically 0.01–0.05/d). For instance, silicate-solubilizing microorganisms have been shown to increase K release rates from phlogopite, illite, and feldspar by >92% relative to sterile controls, with first-order models frequently supplemented by power-law or empirical functions when acceleration is observed. Similar enhancements in apparent rate constants were documented in waste mica leaching experiments inoculated with KSB, confirming the critical role of microbial acidification and chelation. The present study’s finding that k_treatment_ > k_control_ is therefore in excellent agreement with the literature and further validates the effectiveness of the *Burkholderia* strain in promoting potassium release from biotite [48].

### The auxiliary role of amino acids and micro-environmental regulation

A notable observation in this study is the dynamic evolution of extracellular amino acids during the early phase of mineral weathering. Although their absolute concentrations are substantially lower than those of the dominant LMWOAs, the marked accumulation of polar (serine, threonine) and acidic (glutamate, aspartate) amino acids during the initial metabolic explosion (1–5 d) may contribute to establishing the chemical and physical foundation for subsequent biotite degradation (15–30 d) [49,50]. This temporal succession suggests that the early-stage metabolic fingerprint acts as a biochemical primer, working in concert with LMWOAs to initiate an aggressive micro-environment at the mineral surface.

We propose that these amino acids may serve as auxiliary biogenic regulators. The polar side chains could enhance the adhesion of bacterial cells and exopolysaccharides to the biotite surface, while the additional carboxyl groups on acidic amino acids might provide supplementary complexation sites [51,52]. These effects are consistent with the intimate bacterial attachment and localized etch pits observed in SEM images at Day 15 and Day 30 (Fig 8B, C), which provide indirect support for the formation of privileged biofilm-mineral interfaces. Within these micro-zones, the concentrated LMWOAs and H ⁺ are likely to drive sustained lattice destabilization and edge exfoliation [53].

It should be noted that direct experimental evidence for amino acid binding or chelation was not obtained in the present study. Nevertheless, the observed synergy between the early metabolic burst (organic acids + amino acids) and the long-term mineral degradation offers a plausible mechanistic link that extends beyond previously reported proton- and ligand-promoted dissolution processes.

### A conceptual model for KSB-mediated biotite weathering

Based on the integration of chemical, biological, and morphological evidence, we propose a comprehensive model for biotite solubilization by the *Burkholderia* strain:

- Metabolic priming and micro-environment formation: Bacterial cells adhere to biotite basal planes and edges, aided by polar amino acids and exopolysaccharides, triggering an intensive metabolic explosion of H^+^, LMWOAs, and amino acids within the first 120 h.
- Sustained lattice attack: The high-concentration chemical flux established in the priming phase initiates the proton-exchange of interlayer K^+^ and the ligand-promoted complexation of structural Al^3+^ and Fe^3+^.
- Kinetic sustenance: Continuous complexation of framework cations by citrate and malate maintains a state of geochemical undersaturation, driving the continuous expansion of etch pits.
- Physical disintegration: The cumulative effect of structural cation stripping leads to the exfoliation of silicate sheets, culminating in the total collapse of the mineral framework and the irreversible release of K^+^.

### Limitations and future perspectives

We acknowledge that although the early metabolic burst and long-term mineral degradation are strongly correlated through pH dynamics and SEM evidence, direct correlation analysis between metabolite concentrations (1–5 d) and K⁺ release (15–30 d) was limited by differing temporal resolutions. Furthermore, while our results strongly support the auxiliary role of amino acids, direct functional validation (e.g., amino acid supplementation, inhibition assays, or mutant strains) was beyond the scope of the current study. Future research employing such targeted approaches, together with geochemical modeling of the bacteria-mineral interface, will further substantiate and refine the “priming” mechanism proposed here.

## Conclusion

This study systematically investigated the K solubilizing capacity and underlying biochemical mechanisms of a *Burkholderia* strain using biotite as a mineral model. Our findings demonstrate that this strain significantly enhances the mobilization of interlayer K^+^ from biotite, achieving a maximum release of 39.8 mg/L, which is 4.5 times higher than abiotic controls.

The accelerated weathering of biotite is driven by a synergistic “dual-attack” mechanism:

1. Proton-promoted dissolution: The continuous metabolic activity of the live bacteria sustains a low-pH microenvironment (pH ~ 3.8), triggering an effective ion exchange between H^+^ and K^+^.
2. Ligand-promoted complexation: The robust secretion of multidentate low-molecular-weight organic acids (primarily citric and malic acids) facilitates the chelation of structural cations (e.g., Al^3+^, Fe^3+^), thereby destabilizing the aluminosilicate framework and maintaining geochemical undersaturation.

Furthermore, the dynamic accumulation of polar and acidic amino acids (notably serine and glutamate) likely serves as a biogenic auxiliary mechanism, enhancing bacterial-mineral adhesion and interface interactions. Micro-morphological evidence from SEM confirms that these chemical and biological processes culminate in extensive surface roughening, etch pit formation, and edge exfoliation of the mineral lattice.

In summary, this research provides a comprehensive kinetic and structural framework for understanding KSB-mediated mineral weathering. These insights not only advance our knowledge of nutrient cycling at the microbe-mineral interface but also underscore the potential of *Burkholderia* strains as efficient bio-fertilizer candidates for improving K availability in K-deficient soils. Future studies focusing on geochemical modeling and EPS-metal interactions will further refine our understanding of these complex bio-geochemical processes.

## Supporting information

S1 File Supporting information. This document contains S1 Table (physicochemical characteristics of the sampled soil); S2A-S2C Tables (dataset for biotite potassium solubilization experiments including organic acids, amino acids, and soluble potassium/pH variations); S3 Figure (16S-based identification result of strain S1); S4A Figure and S4B Table (mineralogical and physical characterization of the biotite powder); and S5A-S5B Figures (high-resolution SEM images of the bacteria-mineral interface).
(DOCX)
