# Supplementary material for: Unraveling the biochemical and kinetic mechanisms of potassium solubilization from biotite by a Burkholderia strain
Source: PLoS One. 2026 Oct 9;21(10):e0360380. doi: 10.1371/journal.pone.0360380 (PMC13652161; doi:10.1371/journal.pone.0360380)
Supplement: Supplementary file 1 [file pone.0360380.s001.docx]

**S1. Physicochemical properties of the soil sample used in this study.**

**Table** The physicochemical characteristics of the sampled soil

| **Parameters** | **Results** |
| --- | --- |
| pH | 5.63 |
| Water content | 2.7% |
| Organic matter | 23 g/kg |
| Total nitrogen | 600 mg/kg |
| Total phosphorus | 900 mg/kg |
| Water-soluble potassium | 23.93 mg/kg |
| Rapidly available potassium | 302.9 mg/kg |
| Slowly available potassium | 891.3 mg/kg |
| Total potassium | 49.11 g/kg |

**S2. Minimal dataset supporting the results of the biotite potassium solubilization experiments.**

This dataset includes: Concentrations of organic acids and amino acids in the Treatment group (live bacteria + biotite). Soluble potassium (K) concentrations in all groups (CK1, CK2, CK3, and Treatment) at days 0, 5, 10, 15, 20, 25, and 30. pH changes in the Treatment and CK2 groups over the 30-day incubation period.

Detailed experimental design is described in the Materials and Methods section.

**S2A Table.** Organic acid concentrations in the Treatment group during the 30-day leaching experiment.

| Organic acid (mg/L) | 1d | 2d | 3d | 4d | 5d |
| --- | --- | --- | --- | --- | --- |
| Ethanedioic Acid | 1.22 | 1.57 | 1.81 | 1.94 | 2.234 |
| Tartaric Acid | 42.13 | 45.55 | 46.65 | 48.30 | 53.70 |
| Malic Acid | 334.01 | 297.86 | 328.37 | 342.22 | 383.85 |
| Citric Acid | 372.41 | 387.4 | 414.06 | 427.26 | 443.98 |

**S2B Table.** amino acid concentrations in the Treatment group during the 30-day leaching experiment.

| amino acid (nmol/mL) | 1d | 2d | 3d | 4d | 5d |
| --- | --- | --- | --- | --- | --- |
| Glycine | 0.68 | 2.65 | 3.42 | 3.86 | 4.33 |
| Alanine | 0.39 | 1.66 | 2.13 | 2.25 | 1.19 |
| Valine | - | 1.11 | 1.32 | 1.59 | 1.76 |
| Leucine | - | - | - | - | - |
| Serine | 1.36 | 3.39 | 6.85 | 7.91 | 9.14 |
| Threonine | 0.33 | 1.47 | 2.85 | 4.13 | 5.49 |
| Glutamate | - | 2.11 | 2.32 | 2.43 | 2.66 |
| Lysine | - | 0.41 | 0.45 | 0.49 | 0.54 |
| Histidine | - | 0.35 | 0.46 | 0.58 | 0.65 |
| Aspartic acid | 0.16 | 1.65 | 2.16 | 1.88 | 1.76 |

**S2C Table.** Soluble potassium concentrations (mg/L) and pH variation in different treatments (CK1, CK2, CK3, Treatment) at days 0–30.

| **Time(d)** | **CK1** | **CK2** | **CK2_pH** | **CK3** | **Treatment** | **Treatment_pH** |
| --- | --- | --- | --- | --- | --- | --- |
| 0 | 0 | 0 | 5.50 | 0.0 | 0.00 | 6.50 |
| 5 | 0.14 | 1.2 | 5.55 | 0.4 | 1.62 | 6.39 |
| 10 | 0.48 | 3.1 | 5.60 | 0.7 | 3.84 | 5.51 |
| 15 | 1.04 | 4.2 | 5.62 | 1.0 | 4.97 | 4.81 |
| 20 | 6.91 | 7.8 | 5.65 | 1.3 | 9.74 | 4.26 |
| 25 | 7.82 | 14.5 | 5.67 | 1.6 | 22.65 | 4.00 |
| 30 | 8.71 | 18.5 | 5.70 | 1.8 | 39.80 | 3.81 |

**S3 Figure. The result of strain S1 using the 16S-based Identification tool at EZBioCloud.**

**S4. Mineralogical and physical characterization of the biotite powder.**

**S4A Figure.** Energy-dispersive X-ray spectroscopy (EDS) spectrum of the biotite powder used in this study, showing the major elemental composition (K, Mg, Fe, Al, Si, and O).

**S4B Table.** Particle size distribution and BET specific surface area of the prepared biotite powder after grinding and passing through a 1-mm sieve.

| **Parameter** | **Value** |
| --- | --- |
| Particle size ≤ 0.15 mm | 3.70% |
| Particle size 0.15–0.50 mm | 47.86% |
| Particle size 0.50–1.00 mm | 48.44% |
| BET specific surface area | 1.15 m²/g |

**S5. High-resolution scanning electron microscopy (SEM) images of the *Burkholderia* strain S1 attached to biotite surfaces.**

**S5A** **Figure.** SEM micrograph at 5000× magnification showing the colonization and distribution of bacterial cells on the mineral surface.

**S5B** **Figure.** An additional 5000× magnification SEM micrograph highlighting the detailed bacteria-mineral interface. These high-resolution images serve to supplement Figure 8, providing clearer, direct visual evidence of the bacterial attachment during the weathering process.
